# Whole-Genome Sequencing Analysis from the Chikungunya Virus Caribbean Outbreak Reveals Novel Evolutionary Genomic Elements

**DOI:** 10.1371/journal.pntd.0004402

**Published:** 2016-01-25

**Authors:** Kenneth A. Stapleford, Gonzalo Moratorio, Rasmus Henningsson, Rubing Chen, Séverine Matheus, Antoine Enfissi, Daphna Weissglas-Volkov, Ofer Isakov, Hervé Blanc, Bryan C. Mounce, Myrielle Dupont-Rouzeyrol, Noam Shomron, Scott Weaver, Magnus Fontes, Dominique Rousset, Marco Vignuzzi

**Affiliations:** 1 Institut Pasteur, Centre National de la Recherche Scientifique UMR 3569, Viral Populations and Pathogenesis Unit, Paris, France; 2 Institut Pasteur, International Group for Data Analysis, Paris, France; 3 Center for Tropical Diseases and Department of Pathology, University of Texas Medical Branch, Galveston, Texas, United States of America; 4 Institut Pasteur de la Guyane, Laboratoire de Virologie, Centre National de Référence des Arbovirus, Cayenne, French Guiana; 5 Sackler Faculty of Medicine, Tel Aviv University, Tel Aviv, Israel; 6 Institut Pasteur de Nouvelle-Calédonie, Research and Expertise Unit on Dengue and other Arboviruses, Noumea, New Caledonia; University of California, Davis, UNITED STATES

## Abstract

**Background:**

Chikungunya virus (CHIKV), an alphavirus and member of the *Togaviridae* family, is capable of causing severe febrile disease in humans. In December of 2013 the Asian Lineage of CHIKV spread from the Old World to the Americas, spreading rapidly throughout the New World. Given this new emergence in naïve populations we studied the viral genetic diversity present in infected individuals to understand how CHIKV may have evolved during this continuing outbreak.

**Methodology/Principle Findings:**

We used deep-sequencing technologies coupled with well-established bioinformatics pipelines to characterize the minority variants and diversity present in CHIKV infected individuals from Guadeloupe and Martinique, two islands in the center of the epidemic. We observed changes in the consensus sequence as well as a diverse range of minority variants present at various levels in the population. Furthermore, we found that overall diversity was dramatically reduced after single passages in cell lines. Finally, we constructed an infectious clone from this outbreak and identified a novel 3’ untranslated region (UTR) structure, not previously found in nature, that led to increased replication in insect cells.

**Conclusions/Significance:**

Here we preformed an intrahost quasispecies analysis of the new CHIKV outbreak in the Caribbean. We identified novel variants present in infected individuals, as well as a new 3’UTR structure, suggesting that CHIKV has rapidly evolved in a short period of time once it entered this naïve population. These studies highlight the need to continue viral diversity surveillance over time as this epidemic evolves in order to understand the evolutionary potential of CHIKV.

## Author Summary

Chikungunya virus is a re-emerging and rapidly spreading arbovirus that has caused several outbreaks in the last decade with the most recent in the Caribbean islands and the Americas beginning in 2013 infecting over 1 million individuals. The ability to monitor such epidemics would be enhanced by characterizing the viral populations that circulate within infected individuals. To do this, we deep-sequenced viral populations from infected individuals and identified minority variants present at high frequencies, as well as the presence of a novel 3’ untranslated genome region (UTR) structure, a key determinant of chikungunya virus infectivity and evolution never before described in nature. Finally, we genetically engineered an infectious clone from this outbreak strain and established that the novel 3’UTR structure increases viral replication in mosquito cells. Taken together these studies highlight the vast diversity of viral populations in infected individuals, reveal potential novel determinants of chikungunya virus biology, and provide an indispensable tool for future studies involved in viral evolution and adaptation.

## Introduction

Arthropod-borne viruses (arboviruses) pose an eminent threat to public health worldwide and are continuously re-emerging or spreading to uninfected areas. In particular, chikungunya virus (CHIKV) has recently spread to the Americas to cause an estimated 1.7 million cases of severe, debilitating and often chronic arthralgia after roughly 60 years of circulation within Africa and Asia [1] [2] [3] [4]. This raises questions on how CHIKV will spread, evolve, and adapt in new environments in the near future. Previous epidemics of CHIKV have been attributed to adaptive mutations within the viral glycoproteins, allowing the virus to more readily infect the Asian tiger mosquito *Aedes albopictus*, and thus increase its transmission throughout areas of the world harboring this mosquito species. Interestingly, the CHIKV strain that has arrived to the Americas is from the Asian lineage and does not contain these adaptive mutations as of yet. However, both *Aedes aegypti* and *albopictus* are prevalent throughout many parts of North and South America [5] that, along with an enormous naïve human population, give this new strain ample opportunity to undergo adaptive evolution. Using deep-sequencing technologies, we recently characterized the evolution of CHIKV within the mosquito host, where we recapitulated the emergence of previous epidemic variants and identified novel mutations yet to be detected in nature [6]. A survey of the mutant spectra present in human clinical samples, on the other hand, has not yet been performed for CHIKV. Here, we characterize the minority variants directly from human samples, collected between week 52–2013 and week 5–2014, by whole-genome deep sequencing. While no significant consensus changes were observed between these samples collected within a short period of time, our data reveal considerable intra-host genetic diversity. Most importantly, we identify a 3’ untranslated genome region (UTR) duplication that may have been missed by the initial sequencing performed on the ongoing epidemic in the Americas, which seems unique among the circulating CHIKV strains around the world.

## Methods

### Ethics Statement

Samples involved in this study were chosen among human serum specimens received as part of standard diagnostic and expertise activities of the arboviruses National Reference Center for French Departments of the Americas located in French Guiana. The donor samples were rendered completely anonymous and renumbered prior to preparation of extracted RNA for sequencing with only the week of sampling and island of origin retained. Of the 100 samples, 20 gave whole-genome deep sequence coverage and five others (last five in Table 1) gave partial coverage and were retained for analysis, and assigned new IDs as indicated in Table 1.

**Table 1:** Clinical samples used in this study.

Sample^a^	Week-Year Sampled	Island of Origin	RNA copies/ml	Accession Number
M100	52–2013	MART	6.2E+07	LN898093
G100	2–2014	GUA	1.7E+04	LN898094
M101	3–2014	MART	4.7E+04	LN898095
M102	3–2014	MART	2.5E+07	LN898096
G101	3–2014	GUA	1.6E+07	LN898097
G102	3–2014	GUA	2.5E+06	LN898098
G103	3–2014	GUA	3.5E+06	LN898099
M103	3–2014	MART	2.1E+07	LN898100
M104	3–2014	MART	2.8E+07	LN898101
G104	3–2014	GUA	2.2E+07	LN898102
G105	3–2014	GUA	3.0E+06	LN898103
M105	3–2014	MART	9.4E+06	LN898104
M106	3–2014	MART	3.3E+06	LN898105
M107	3–2014	MART	1.3E+07	LN898106
M108	3–2014	MART	8.6E+05	LN898107
M109	4–2014	MART	4.8E+07	LN898108
M110	4–2014	MART	4.5E+06	LN898109
G106	5–2014	GUA	8.7E+07	LN898110
G107	5–2014	GUA	3.6E+06	LN898111
M111	3–2014	MART	8.0E+06	LN898112
M112	3–2014	MART	2.1E+06	n/a
M113	4–2014	MART	7.6E+04	n/a
M114	4–2014	MART	2.8E+04	n/a
M115	3–2014	MART	1.5E+06	n/a
M116	3–2014	MART	1.2E+03	n/a

^a^Individual in which infectious clone was derived.

MART = Martinique, GUA = Guadeloupe, n/a—not submitted to bank, incomplete genome coverage

### Selection of Clinical Samples

100 human sera positive for CHIKV qRT-PCR were randomly selected amongst (1) those sampled between week 52 of 2013 and week 5 of 2014 around the beginning of epidemic phase in the French Caribbean islands, and (2) those having a high viral load (mostly between 10^6^ and 10^7^ copies/ml, even if some lower viral loads were added to examine whether sampling bias with respect to viral load had occurred (**Table 1**). One third of these samples were from Guadeloupe and two thirds from Martinique. The consensus sequences of the 20 whole-genome samples were deposited in the European Nucleotide Archive, with the accession numbers indicated in Table 1, and accessible at http://www.ebi.ac.uk/ena/data/view/LN898093-LN898112.

### Deep-Sequencing Analysis

Total RNA from patient serum was isolated by Trizol (Sigma) extraction following the manufacturer’s protocol, resuspended in nuclease free water, and used directly for cDNA synthesis using the Maxima H Minus First Strand cDNA Synthesis Kit (Thermo Scientific) with random hexamers. Following cDNA synthesis, approximately 2 kb amplicons of the CHIKV genome were amplified by Phusion DNA polymerase using the primers designed based on the published St. Martin CHIKV strain CNR20235 (**Table 2**) (http://www.european-virus-archive.com/article147.html). Amplicons were subsequently purified via a nucleospin PCR purification kit (Macherey-Nagel), quantified by picogreen, and fragmented as described previously [6]. Sequences were obtained with an Illumina NextSeq500 machine and aligned against the CNR20235 reference sequence using the ViVAn pipeline [7], which differentiates statistically significant variants from total SNPs identified within reads. For example, patient 1, amplicon 1 presented 2557 SNPs in the quality filtered reads along the 871 nucleotide sites sequenced, with the lowest frequencies at 0.00001. ViVan statistical analysis further reduced these SNPs to 1188 with the lowest frequency at 0.0001 for an average read coverage of 80,000X. We set an additional, conservative cut-off of a minimum of 3,000X coverage and 0.001 frequency, bringing the total SNPs in this sample to 564. Average coverages were above 70,000X for all samples. All samples had similar profiles to the example given above, with no apparent outliers, with 95–100% of all possible SNPs represented in quality filtered reads, 36–48% of SNPs in ViVan filtered data and 16–23% of SNPs above the conservative cut-off. Variants with a frequency above 0.5 (50% of the total population) were considered consensus changes and were added to the CNR20235 reference sequence. The consensus sequences obtained from the 20 whole-genome samples were deposited in the European Nucleotide Archive with accession numbers listed in **Table 1**. To align “total” and “unique” reads an in-house pipeline was used. The reads were trimmed to remove low-quality bases using fastq-mcf [8] and aligned with bwa-mem [9] to an artificial reference genome consisting of the two references genomes.

For tissue culture passaged virus deep-sequencing, human sera were placed directly on Vero or C6/36 cells and supernatants were collected three days post-infection for C6/36 cells or at cell death for Vero cells. Viral RNA was extracted and analyzed as described above.

**Table 2:** Primers used to PCR amplify the chikungunya virus genome.

Primer Name	Sequence (5’—-3’)	Genome Region
Primer 1 Forward	CACGTAGCCTACCAGTTTCTTA	5’ UTR–nsP1
Primer 2 Reverse	ATGGAACACCGATGGTAGGTG		
Primer 3 Forward	AACCCCGTTCATGTACAACGC	nsP1–nsP2
Primer 4 Reverse	CGGCATGTTGTACTCATTCG		
Primer 5 Forward	CGAATTCGACAGCTTTGTAG	nsP1–nsP2
Primer 6 Reverse	GCACATGATGTCCGTTTATC		
Primer 7 Forward	GACCAAGACTGAAAGTTGTAC	nsP2–nsP3
Primer 8 Reverse	CCACATAGTATGTATCTCTGC		
Primer 9 Forward	GCGTACTGGGACGTAAGTTTA	nsP2–nsP3
Primer 10 Reverse	GGACGCACTCTCCTGGAGTTTC		
Primer 11 Forward	CTGTACGGGAAGTGAGTATGAC	nsP3–nsP4
Primer 12 Reverse	CATACCGGATTTCATCATAGC		
Primer 13 Forward	GGAGACGCCGTTTTAGAAACG	nsP4–capsid
Primer 14 Reverse	CGCTTGAAGGCCAATTTGGCC		
Primer 15 Forward	GCAGAGAGAGAATGTGCATG	Capsid–E2
Primer 16 Reverse	CCGCTTTAGCTGTTCTAATGC		
Primer 17 Forward	GGAACTACCTTGCAGCACGTAC	E2–E1
Primer 18 Reverse	GGCGTTAGTCATCGAGTGCAC		
Primer 19 Forward	GTACAGCAGAGTGTAAGGACCA	E1–3’UTR
Primer 20 Reverse	CATATACCTTCTTACCTAC		
Primer 21 Forward	GAACATGCCTATCTCCATCGAC	E1–3’UTR
Primer 22 Reverse	AACATCTCCTACGTCCCTATGG		

### Phylogenetic Alignment and Analysis

Full-length CHIKV sequences were aligned using the CLUSTAL W program [10]. Once aligned, the program Model Generator [11] was used to identify the optimal evolutionary model that best described our sequence dataset. Akaike information criteria and hierarchical likelihood ratio test indicated that the GTR + Γ + I model best fit the sequence data. Maximum-likelihood phylogenetic trees were constructed under the GTR + Γ + I model using software from the PhyML program [11]. As a measure of the robustness of each node, we used an approximate Likelihood Ratio Test (aLRT), which demonstrates that the branch studied provides a significant likelihood against the null hypothesis that involves collapsing that branch of the phylogenetic tree but leaving the rest of the tree topology identical. aLRT was calculated using three different approaches: (a) minimum of Chi square-based calculations; (b) a Shimodaira-Hasegawa-like procedure (SH-like) [12] [13], which is non-parametric, and (c) a combination of both (SH-like and the minimum Chi square-based calculations), which is the most conservative option for these calculations. In addition, the bootstrap method was also used.

### Construction of Caribbean Strain Infectious Clone

Patient serum was first inoculated on the *Ae*. *albopictus* mosquito cell line C6/36 and CHIKV obtained was subsequently amplified on Vero cells to generate a working viral stock. Viral RNA was isolated by Trizol extraction and cDNA was synthesized as described above. The infectious clone was constructed using four PCR amplicons generated by Phusion DNA polymerase using the primers in **Table 3**and subcloned into the plasmid containing the published Indian Ocean Lineage (IOL) infectious clone [14] using common restriction sites. In brief, amplicon one was subcloned into the BamHI and AgeI restriction sites of the IOL infectious clone also generating a unique AgeI restriction site in the Caribbean CHIKV infectious clone sequence. The BamHI site was then removed by site-directed mutagenesis. Amplicon two was subcloned between the two AgeI restriction sites, followed by the subcloning of three into the 3’ AgeI and XhoI restriction sites. Finally, amplicon four was subcloned into the XhoI and NotI restriction sites. Each cloning precursor was Sanger sequenced and the final clone was Sanger sequenced in full.

**Table 3:** Primers used to construct Caribbean chikungunya virus infectious clone.

Primer Name	Sequence (5’—-3’)
BamHI Forward	GATTAATAACCCATCATGGATCCTG
AgeI Reverse	GTTGTAAATGGCCTGGACCGGTGTC
AgeI Forward	GGTATATATTCTCGTCGGACACCGG
AgeI2 Reverse	GGCTTCTTTTTCTTTTGAACCGGTT
AgeI2 Forward	GCCCCCCAAAAAGAAACCGGTT
XhoI Reverse	GTTACCCCACGTGACCTCGAGCC
XhoI Forward	GTTAACCGTGCCGACTGAGGGGCTCG
PolyANotI Reverse	GAGGATGCATTGCGGCCGCTTTTTTTTTTTTTTTTTTTTTTTTTTTTTTTTTTTTTTTTTGAAATATTAAAAACAAAATAAC
BamHI removal Forward	CCCATCATGGATtCTGTGTACGTGGATATAG
BamHI removal Reverse	CTATATCCACGTACACAGaATCCATGATGGG

### Cell Culture and Viruses

Baby Hamster Kidney (BHK-21) cells and Vero cells were maintained in Dulbecco’s Modified Eagle’s Media (DMEM) supplemented with 10% fetal calf serum and 1% penicillin/streptomycin (P/S) at 37°C with 5% CO_2_. *Ae*. *albopictus* cells (C6/36 and U4.4) were maintained in Leibovitz L-15 media supplemented with 10% fetal bovine serum, 1% P/S, 1% nonessential amino acids, and 1% tryptose phosphate broth at 28°C and 5% CO_2_.

Working viral stocks from the Caribbean infectious clone was generated as described previously [14]. The Caribbean strain infectious clone lacking the 3’UTR duplication was commercially synthesized and supplied by the laboratory of Andres Merits. The CHIKV strain NC/2011-58 (accession # HE806461) was a gift from the Institut Pasteur–New Caledonia. All viruses were passaged once over BHK cells to obtain a working viral stock. Viral titers were determined by plaque assay on Vero cells as previously described [14]. The strains from Mexico, Dominican Republic and Trinidad used to confirm the presence of the 3'UTR duplication were obtained from Scott Weaver and Rubing Chen.

### Viral Growth Curves

Mammalian cells (BHK-21 and Vero) and insect cells (C6/36 and U4.4) were infected with each virus at an MOI of 0.1 in infection media (DMEM containing 0.2% bovine serum albumin, 1 mM HEPES pH 7.4, and 1% P/S) for one hour at 37°C for mammalian cells and 28°C for insect cells. Virus was subsequently removed and cells were washed twice with phosphate buffered saline (PBS) and complete media was added. Aliquots of the viral supernatant were taken at the indicated time points and viral titers were determined by plaque assay as described previously [14].

## Results

**Fig 1:**
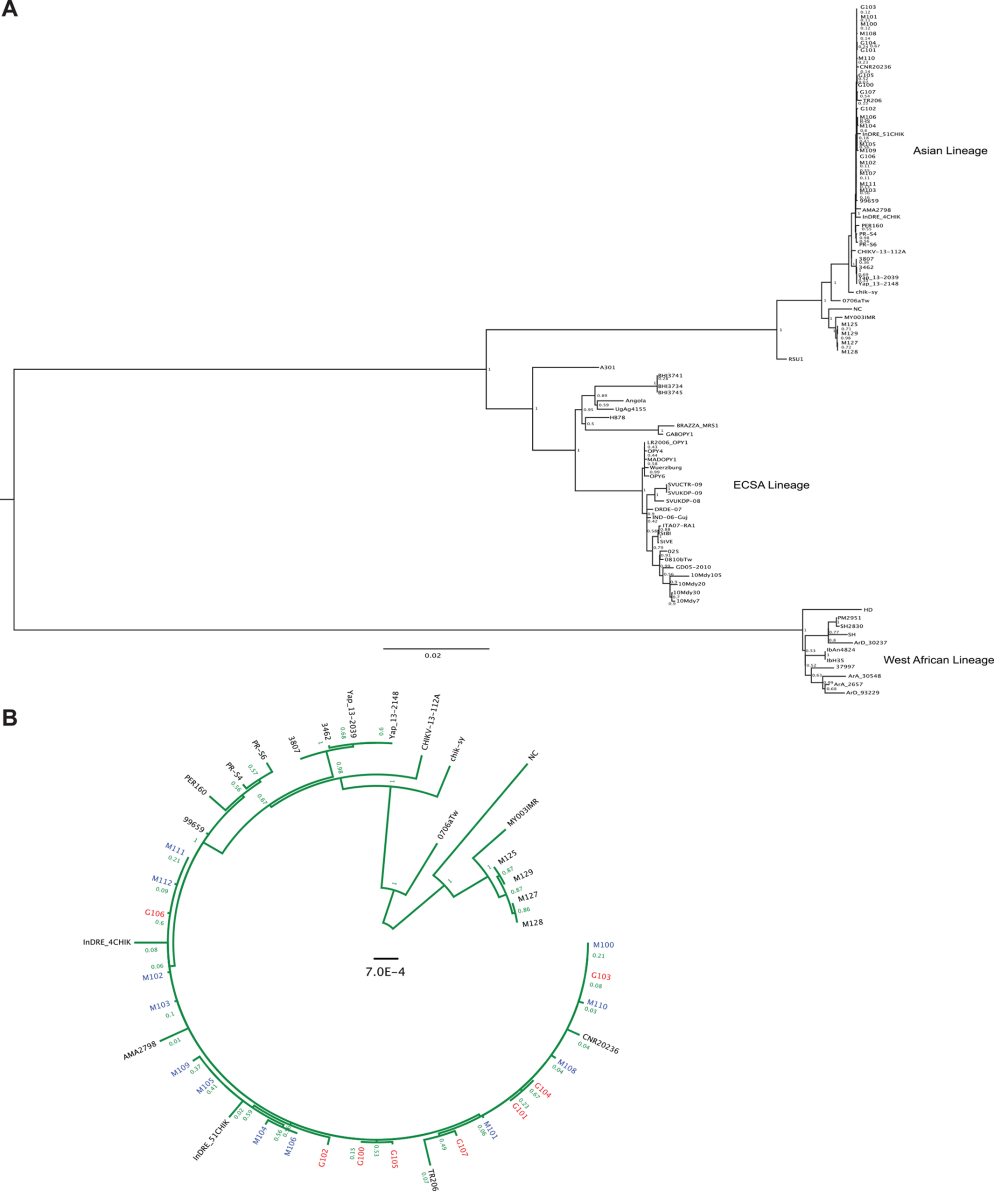
Phylogenetic analysis of chikungunya virus human samples from Martinique and Guadeloupe. A. Maximum likelihood phylogenetic tree of chikungunya virus strains using complete genome sequences. Scale bar represents genetic distance. Numbers at the branches show aLRT values (1 = 1000). Genotypes are indicated on the right. B. Maximum likelihood phylogenetic tree based on the Caribbean outbreak cluster with additional Asian strains of chikungunya virus using full genome sequences. Scale bar denotes genetic distance. Numbers at the branches show aLRT values (1 = 1000). Strains from Martinique are in blue and those from Guadeloupe are in red.

To analyze the viral diversity present within human hosts infected during the current CHIKV outbreak in the Americas, we deep-sequenced 25 viral strains from sera of infected patients from Martinique and Guadeloupe (**Table 1**). The samples, consisting of approximately 70% from Martinique, were taken between weeks 52–2013 and week 5–2014 and represented the same proportion of patients who were diagnosed as CHIKV positive during this time. By using our deep sequencing data we assembled the consensus sequences obtained from each patient to determine the degree of genetic variability of these strains by phylogenetic approaches. These sequences were aligned with 63 full-length CHIKV strains isolated elsewhere, representing all major CHIKV lineages. Subsequently, maximum likelihood phylogenetic trees were constructed. The results of our analysis were in agreement with previous studies [1] [15][16], placing these viruses into the Asian Lineage of CHIKV and to cluster with the St. Martin strain CNR20236 (**Fig 1A and 1B)**.

Furthermore, deep-sequencing analysis identified a variety of unique high-frequency intra-host minority variants (at greater than 0.5% frequency) in infected individuals, as well as five synonymous consensus sequence differences with respect to the initially reported strain from St. Martin, which were common to all patients (nsP2 position 2716, A>G; nsP3 position 4507, C>A; position 4513, A>G; and the 3’UTR position 11952, C>T; position 11953, G>A) (**Table 4**, bold).

**Table 4:** Frequency of significant mutations (>0.001) identified in patient samples.

GenomeRegion	nt Position^a^	nt Change	AA Change^b^^,^^c^	M100	G100	M101	M102	G101	G102	G103	M103	M104	G104	G105	M105	M106	M107	M108	M109	M110	G106	G107	M111
5’UTR	53	A>T		0.106	0.089	0.077	0.086	0.081	0.070	0.078	0.088	0.066	0.072	0.071	0.004	0.004	0.003	0.003	0.003	0.003	0.003	0.002	0.003
nsp1	476	T>A	C134S	0.101	0.098	0.090	0.098	0.097	0.106	0.094	0.100	0.107	0.090	0.102	0.017	0.017	0.012	0.010	0.013	0.011	0.012	0.010	0.015
nsp1	479	T>C	syn>L						0.988			0.999			0.999	0.985		0.061	0.999	0.010		0.006	
nsp1	633	A>C	N186T	0.010	0.011	0.010	0.010	0.011	0.009	0.011	0.008	0.103	0.008	0.009	0.004	0.004	0.049	0.005	0.006	0.005	0.013	0.005	0.004
nsp1	667	A>T	syn>T	0.071	0.064	0.078	0.040	0.038	0.044	0.041	0.062	0.192	0.040	0.041	0.011	0.012	0.015	0.004	0.005	0.004	0.006	0.005	0.012
nsp1	734	G>C	G220R									0.127											
nsp1	809	T>G	S245A	0.100	0.097	0.089	0.084	0.076	0.092	0.096	0.084	0.079	0.073	0.102	0.051	0.052	0.029	0.041	0.049	0.037	0.046	0.046	0.051
nsp1	864	T>A	V263E	0.113	0.009						0.019	0.016	0.007	0.005	0.001	0.001	0.003	0.001	0.004	0.002	0.002	0.002	
nsp1	1112	C>T	L346F														0.120						
nsp1	1525	C>T	syn>Y									0.994			0.995	0.996		0.107	0.995				
nsp1	1527	G>A	S484N															0.100					
nsp1	1571	G>A	E499K														0.188				0.004		
nsp2	2008	C>T	syn>H														0.158						
nsp2	2038	G>A	syn>S	0.002	0.971							0.002		0.981									
nsp2	2639	A>T	I320F	0.011	0.023	0.012	0.011	0.013			0.010	0.103	0.014	0.014	0.009	0.006	0.005	0.004	0.005	0.007	0.005	0.005	
nsp2	2672	C>T	R331C									0.110		0.002					0.002				
**nsp2**	**2716**	**A>G**	**syn>T**	**0.997**	**0.997**	**0.995**	**0.994**	**0.993**	**0.995**	**0.998**	**0.998**	**0.997**	**0.996**	**0.994**	**0.997**	**0.998**	**0.998**	**0.998**	**0.997**	**0.997**	**0.998**	**0.997**	**0.997**
nsp2	2788	T>A	syn>I	0.123	0.094	0.133	0.098					0.073	0.087	0.090	0.022	0.021	0.021	0.014	0.012	0.017	0.014	0.013	
nsp2	2962	T>C	syn>S																			0.999	
nsp2	3063	T>G	I461S	0.065	0.058	0.052	0.053					0.119		0.069	0.018	0.018	0.017	0.022	0.017	0.017	0.022	0.018	
nsp2	3196	T>A	syn>A	0.005	0.006	0.004	0.006					0.004		0.008	0.002	0.002	0.156	0.002	0.002	0.003	0.002	0.002	
nsp2	3519	T>A	V613E	0.116	0.129	0.056	0.047					0.096			0.010	0.009	0.014	0.008		0.007	0.008	0.008	
nsp2	3591	T>A	L637H	0.002		0.003	0.004							0.005	0.001	0.002	0.002	0.002			0.131	0.001	
nsp2	3996	G>A	G772D																0.504				
nsp3	4219	T>A	S48R	0.031	0.032	0.037	0.037	0.028	0.033		0.038	0.160	0.180	0.036	0.006	0.005	0.006	0.006	0.003	0.007	0.005	0.006	0.005
nsp3	4221	C>T	A49V	0.136								0.002											
nsp3	4334	G>A	A88T										0.001									0.144	
nsp3	4409	G>C	G113R																0.671				
nsp3	4479	C>T	T135M										0.002	0.001	0.463								
**nsp3**	**4507**	**C>A**	**syn>R**	**0.994**	**0.995**	**0.994**	**0.992**	**0.995**	**0.993**	**0.999**	**0.993**	**0.994**	**0.994**	**0.994**	**0.999**	**0.998**	**0.998**	**0.998**	**0.997**	**0.997**	**0.999**	**0.997**	**0.997**
**nsp3**	**4513**	**A>G**	**syn>K**	**1.000**	**0.999**	**0.999**	**0.999**	**0.998**	**1.000**	**0.999**	**0.998**	**0.999**	**0.999**	**0.999**	**0.999**	**0.999**	**0.999**	**0.999**	**0.999**	**0.999**	**1.000**	**0.999**	**0.999**
nsp3	4605	G>A	R178Q						0.957										0.003				0.001
nsp3	4606	C>A	R178Q						0.959														
nsp3	4839	C>T	S255F									0.991											
nsp3	4864	T>A	syn>L	0.021	0.015	0.015	0.008				0.020	0.018	0.011	0.017	0.004	0.002	0.003	0.003	0.004	0.005	0.999	0.089	0.003
nsp3	5090	T>C	S340P			0.987																	
nsp3	5151	A>C	D359A	0.047	0.033	0.061	0.093	0.058	0.122		0.195	0.045	0.066	0.055	0.021	0.054	0.039	0.023	0.033	0.026	0.029	0.027	0.031
nsp3	5179	A>C	E368D	0.041	0.016	0.058	0.106	0.065	0.133		0.075	0.050	0.034	0.052	0.021	0.034	0.023	0.020	0.024	0.016	0.020	0.016	0.019
nsp3	5190	A>C	D359A	0.031	0.017	0.041	0.080	0.045	0.114		0.017	0.039	0.043	0.043	0.022	0.034	0.022	0.019	0.025	0.016	0.017	0.017	0.019
nsp3	5221	T>A	syn>L	0.075	0.063	0.105	0.071	0.074	0.039		0.068	0.067	0.075	0.063	0.006	0.005	0.007	0.005	0.005	0.005	0.004	0.005	0.006
nsp3	5298	T>A	V408E	0.027	0.018	0.032	0.020		0.151		0.022	0.025	0.020	0.025	0.004	0.003	0.004	0.004	0.005	0.004	0.004	0.004	0.003
nsp3	5305	T>A	C410Stop	0.037	0.027	0.042	0.041		0.115		0.026	0.021	0.022	0.022	0.005	0.004	0.004	0.004	0.004	0.004	0.004	0.004	0.002
nsp3	5315	G>A	E415K															0.745					
nsp3	5325	T>A	I417K	0.025	0.034	0.026	0.119		0.036		0.023	0.017	0.023	0.018	0.010	0.012	0.010	0.003	0.005	0.004	0.003	0.004	0.007
nsp3	5334	T>A	M420K	0.023	0.024	0.020	0.121		0.033		0.026	0.020	0.019	0.020	0.004	0.004	0.005	0.004	0.004	0.003	0.004	0.003	0.005
nsp3	5376	T>C	V435A																0.224		0.003		
nsp3	5564	C>T	syn>L																0.227				
nsp4	5999	T>C	S116P	0.008	0.008		0.001	0.001												0.065			
nsp4	6075	T>C	I141T				0.004								0.105				0.017		0.002		
nsp4	6687	T>C	L345P															0.179					
nsp4	6767	A>C	I372L		0.105																		
nsp4	6805	G>A	syn>A	0.078	0.086	0.076	0.057	0.060	0.017	0.120	0.065	0.004	0.002	0.001	0.001								
nsp4	6864	T>C	I404T								0.969												
nsp4	6975	T>A	L441Stop															0.179					
nsp4	7076	T>G	S475A	0.072	0.069	0.079	0.080	0.074	0.625	0.079	0.078	0.075	0.070	0.063	0.030	0.013	0.015	0.013	0.013	0.010	0.013	0.010	0.015
nsp4	7159	T>C	syn>A	0.063	0.051	0.063	0.100	0.078		0.090	0.062	0.076	0.082	0.090	0.017	0.016	0.020	0.015	0.020	0.015	0.014	0.018	0.012
E3	8492	A>G	Q52R												0.143								
E2	8892	T>C	syn>I		1.000		1.000		1.000		1.000				0.998		1.000		0.998		1.000		
E2	9690	G>A	syn>G	0.049			0.068				0.176	0.989			0.069		0.072				0.058		
E1	10335	T>A	F118L	0.097	0.114	0.084	0.115	0.106	0.090	0.091	0.114	0.096		0.014	0.012	0.014	0.012	0.014	0.011	0.010	0.011	0.015	
E1	11046	T>C	syn>S	0.002																		0.924	
3UTR	11302	C>T					0.984																
3UTR	11311	T>C						0.979	0.001				0.997										
3UTR	11416	T>A		0.015	0.005	0.005	0.006	0.003	0.002	0.008	0.005	0.011	0.002	0.002	0.003	0.001	0.004	0.004	0.005	0.996	0.003	0.006	
3UTR	11525	C>T		0.412			0.016					0.003			0.006		0.004	0.464	0.004	0.474	0.021	0.018	
**3UTR**	**11775**	**C>T**		**0.955**		**0.980**	**0.966**	**0.897**	**0.997**	**0.946**	**0.957**	**0.965**			**0.996**		**0.995**	**0.872**	**0.989**	**0.892**	**0.982**	**0.921**	
**3UTR**	**11776**	**G>A**		**0.995**		**0.997**	**0.990**	**0.977**	**0.997**	**0.993**	**0.997**	**0.993**			**0.997**		**0.997**	**0.890**	**0.996**	**0.916**	**0.994**	**0.946**	
3UTR	11791	C>T		0.362													0.586	0.624	0.595	0.640		0.593	

^a^ nt = nucleotide

^b^ AA = amino acid

^c^ syn = synonymous

Bold print indicates five novel synonymous sequences changes. The table shows any mutation present above 0.1 in at least one individual, and any mutation above 0.001 found in more than one individual.

We then evaluated the overall genetic variability of the outbreak strain across the entire CHIKV genome. With the exception of the five consensus changes, yielding a frequency of 1, we found a number of high-frequency minority variants scattered throughout the genome; yet the majority were unique to individual patients, suggesting they did not mediate significant adaptation at the population level. In particular, these variants were found primarily in the nonstructural proteins and 3’UTR with only several variants present in the structural genes. When we analyzed low-frequency minority variants we found a diverse population containing variants with frequencies ranging from as much as 30% to less than 0.1% of the population.

**Fig 2:**
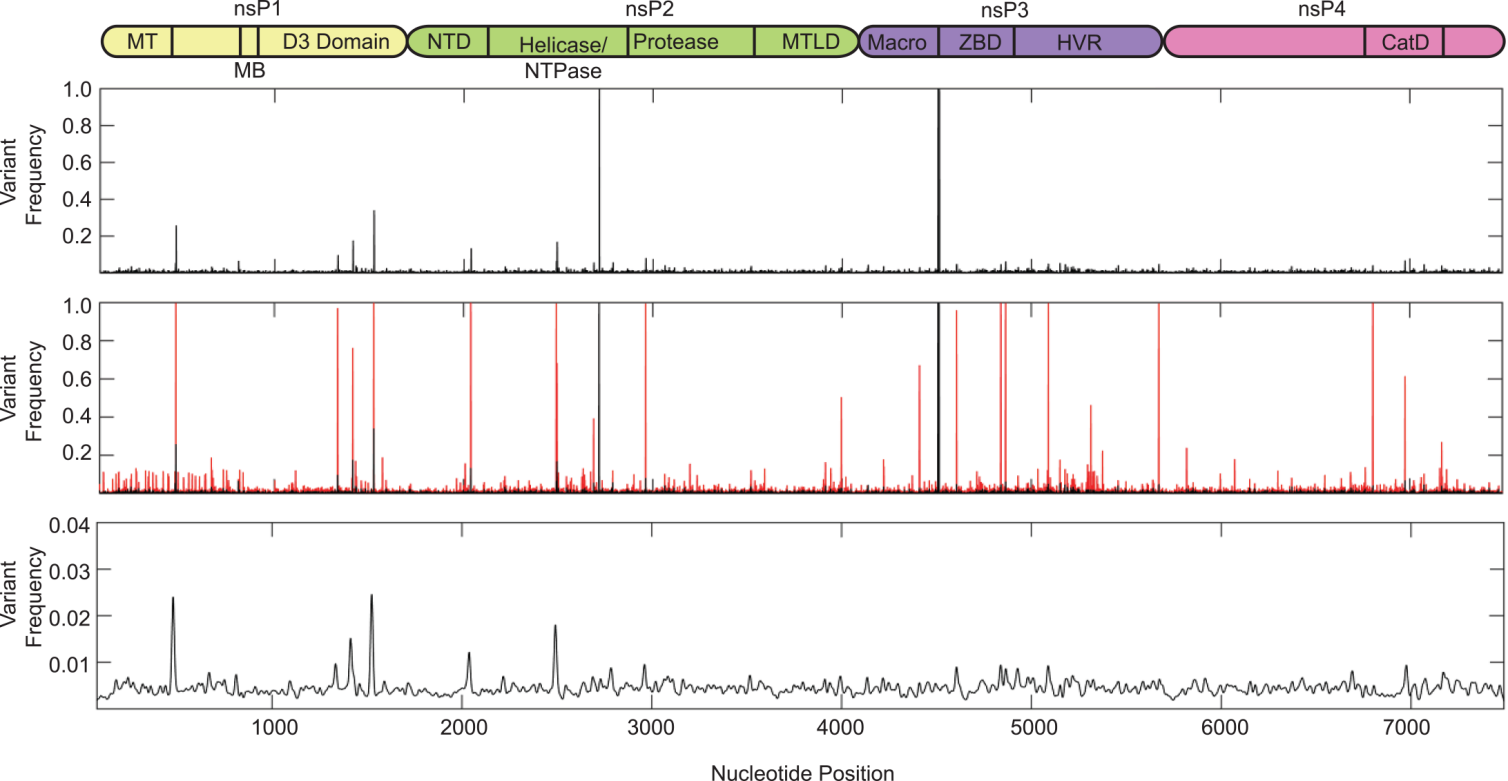
Minority variant analysis of chikungunya virus nonstructural proteins. Schematic representation of the chikungunya virus nonstructural proteins is represented above the graph. Nonstructural domains represent the following: nsP1, nonstructural protein 1, genome nucleotides 77–1681, MT = methyltransferase domain (amino acids 1–170), MB = membrane binding domain (amino acids 171–300); nsP2, nonstructural protein 2, genome nucleotides 1682–4072, NTD = N-terminal domain, MTLD = methyltransferase like domain; nsp3, nonstructural protein 3, genome nucleotide 4073–5653, ZBD = zinc binding domain, HVR = hypervariable region (amino acids 328–530); nsP4, nonstructural protein 4, genome nucleotides. 5654–7489, CatD = catalytic domain (amino acids 365–479). Variants expressing a frequency of greater than 0.001 were used for analysis. Top graph represent the mean variant frequency over all samples. Middle graph represents the mean frequency (black) and variant range (red) over all samples. Bottom graph represents mean variant frequency smoothed with a Gaussian kernel.

**Fig 3:**
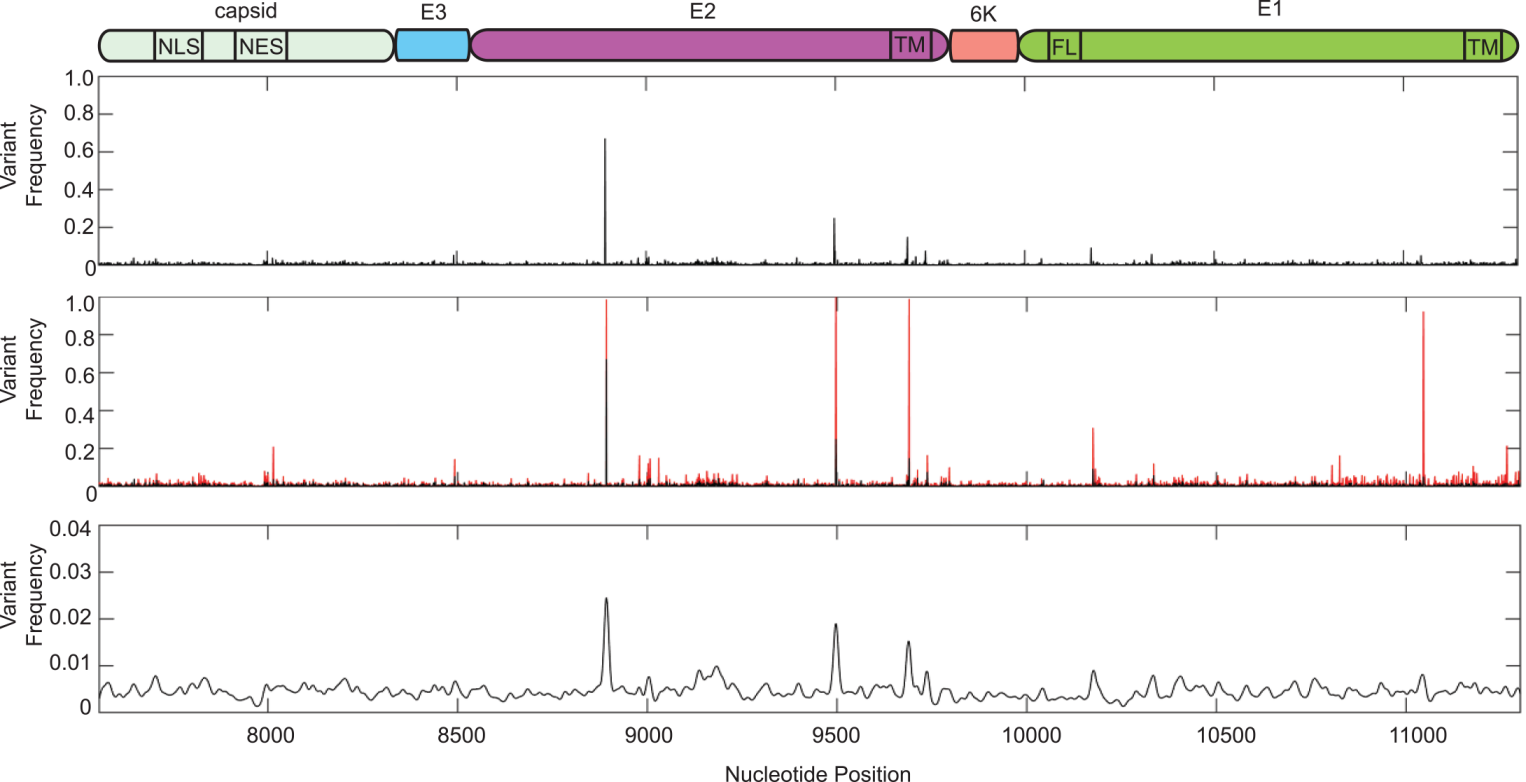
Minority variant analysis of chikungunya virus structural proteins. Schematic representation of the chikungunya virus structural proteins is represented above. Capsid, genome nucleotides 7555–8337, NLS = nuclear localization signal (amino acids 60–99), NES = nuclear export signal (amino acids 143–155); E3, genome nucleotides 8338–8529; E2, genome nucleotides 8530–9801, TM = transmembrane domain (amino acids 363–391); 6k, genome nucleotides 9802–9981; E1, genome nucleotides 9982–11301, FL = fusion loop (amino acids 83–101), TM = transmembrane domain (amino acids 413–436). Variants expressing a frequency of greater then 0.001 were used for analysis. Top graph represent the mean frequency over all samples. Middle graph represents the mean frequency (black) and variant range (red) over all samples. Bottom graph represents mean variant frequency smoothed with a Gaussian kernel.

We next analyzed the specific variants in each gene (**Figs 2**and **3**). We found that the nonstructural proteins presented many more variants (56 at a frequency of at least 10% of the population) than the structural proteins (5 variants at a frequency above 10% of the population). In particular, within nsP1, which functions as the methyltransferase and is necessary for RNA replication, we observed eight amino acid changes and three synonymous changes in individual patients and localized variation concentrated in the methyltransferase and D3 domains. Nsp2 is a multifunctional protein serving as the viral helicase, protease, and NTPase. Although we observed considerable variation scattered across nsP2, this protein contained six synonymous and six amino acid changes including one mutation (G772D) present at roughly 50% of the viral population in one individual. The nsP3 gene, which encodes a phosphoprotein required for RNA replication, contained the largest number of minority variants, with 23 total changes. These include only five synonymous changes and 18 coding changes (**Fig 2**and **Table 4**), with several minority variants making up a considerable portion of the viral population. We noted the mutations of several serine residues in nsP3 (S48R, S255F, and S340P) that were present at nearly 100% in one individual that could change the phosphorylation state of the protein, as well as several charge changes (G113R, R178Q and E415K) that may have functional roles in RNA binding or viral replication. The viral RNA dependent RNA polymerase, nsP4, contained 10 sequence changes with three synonymous and seven coding changes. Interestingly, one patient presented a stop codon (L441Stop) at 63% of the viral population that is coupled with the R178Q mutation of nsP3 mentioned above. It is possible that these mutations may function together to oblate the large amount of truncated nsP4 in this individual.

In contrast, the structural genes presented fewer numbers and frequencies of minority variants, with only two nonsynonymous variants in E3 and E1 genes, with frequencies between 10 and 14 percent of the viral population (**Fig 3**). Given the role of the viral glycoproteins E2 and E1 in viral evolution and transmission, we looked at the overall genetic variation within these two proteins. Here, we found portions of the protein involved in protein-protein interactions (Domains I and II of E1, as well as the E2 and E1 stem domains) and the transmembrane domains required for membrane binding to contain variation. Interestingly, regions flanking the fusion-loop (amino acids 83–101) of E1 contained more variation then the fusion-loop itself indicating that changes directly in the fusion loop are poorly tolerated. Importantly, no samples contain any significant levels of previously observed vector-adaptive mutations such as E2 L210Q, E1T98A, or E1 A226V, which could potentially facilitate dissemination of this virus in *Aedes albopictus* mosquitoes [17].

**Fig 4:**
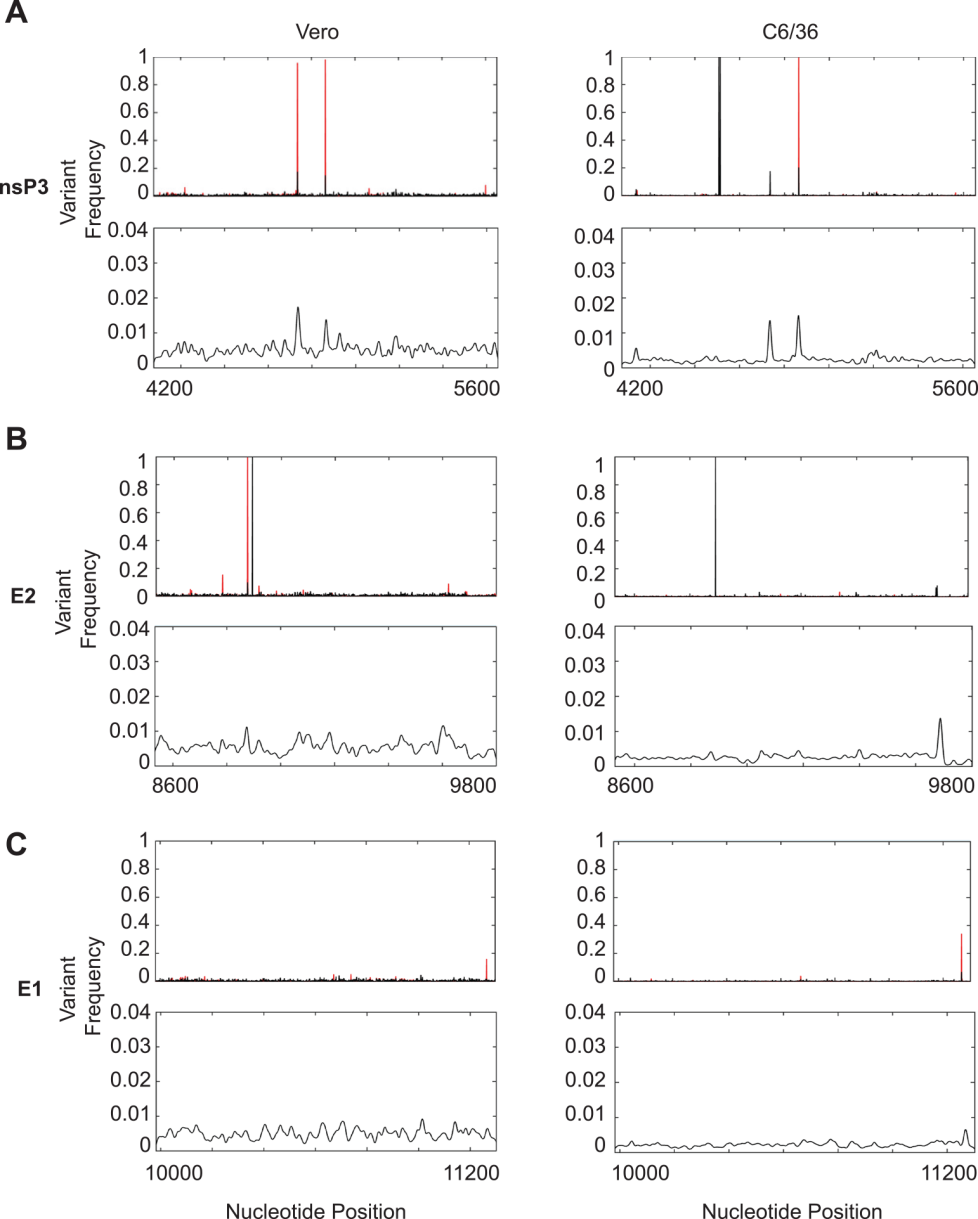
Comparison of minority variants present in either mammalian or insect tissue culture passaged viral stocks. A. Frequency of variants present in nsp3 (nucleotides 4073–5653). B. Frequency of variants present in E2 (nucleotides 8530–9801). C. Frequency of variants present in E1 (nucleotides 9982–11301). Top graphs represent the mean variant frequency (black) and variant frequency range (red) over each sample. Bottom graphs represent mean low-level variant frequency (0.001–0.04) smoothed with a Gaussian kernel.

In addition to the variation within serum samples, we also addressed how minority variants change as they are passaged through tissue culture, a technique that is commonly used to amplify viral stocks from low-titer human samples. By passaging five different individual sera once through mammalian and insect cells, we found the number of higher-frequency variants dropped considerably from roughly 60 variants in the human samples to 15 in the tissue culture passages (**Table 5**). Of the 15 variants, the six consensus sequence changes were maintained in insect cells; however, passaging through mammalian cells removed the two consensus changes in nsP3 (positions 4507 and 4513). Furthermore, passaging virus through tissue culture cells also identified five novel variants not found in the human samples. These included two synonymous changes in E2 (position 8874, C>T) and E1 (position 10104, G>A), as well as three coding changes in nsP2 (G460S, 37% of the population in mammalian cells), nsP4 (L455M, 95% of the population in insect cells), and E1 (G274V, 95% of the population in mammalian cells) in unique sera passages. When we compared the variants between serum and tissue cultured passages of the same samples, we found that the high-frequency variants present in the sera were indeed maintained over passaging. Finally, passaging each virus a single time through mammalian cells maintained more diversity than passaging virus once through insect cells (**Fig 4,** bottom graphs in each panel), something that has been seen previously when looking at viral adaptation between such disparate hosts [14]. Taken together, these data shed light on variable hot spots within the CHIKV genome, identified novel variants circulating at high frequency in individuals, and suggests that if wildtype-like population diversity is desired, it may be best to passage viral strains through highly-permissive mammalian cell lines.

**Table 5:** Frequency of significant mutations (>0.001) identified in tissue culture passaged viral populations.

GenomeRegion	nt Position	nt Change	AA Change	C6/36-1	C6/36-2	C6/36-3	C6/36-4	C6/36-5	Vero-1	Vero-2	Vero-3	Vero-4	Vero-5
nsp1	479	T>C	syn>L	0.010		1.000		1.000					
nsp1	1525	C>T	syn>Y	0.006				0.996		0.973		0.978	0.012
nsp2	2716	A>G	syn>T	0.996	0.999	0.997	0.997	0.997	0.999	0.999	0.999	0.999	0.999
nsp2	3059	G>A	G460S						0.371				
nsp3	4507	C>A	syn>R	0.998	0.998	0.998	0.997	0.998					
nsp3	4513	A>G	syn>K	0.999	0.999	0.999	0.999	0.996					
nsp3	4864	T>A	syn>L	0.007	0.003	0.004	0.999	0.003	0.012	0.011	0.990	0.009	0.011
nsp4	7016	C>A	L455M			0.951	0.020	0.081					
E2	8874	C>T	syn>F	0.005		0.002	0.002	0.002	0.990				
E2	8892	T>C	syn>I	0.999		0.998		0.998	1.000	1.000	1.000	0.999	1.000
E1	10104	G>A	syn>T	0.979		0.002	0.003	0.004	0.002	0.002	0.003	0.002	0.968
E1	10802	G>T	G274V	0.044	0.041	0.096	0.093	0.088			0.959		
3UTR	11775	C>T		0.978			0.952				0.985		0.981
3UTR	11776	G>A					0.999				0.995		0.994
3UTR	11791	C>T		0.359			0.291				0.499		0.539

nt = nucleotide, AA = amino acid, syn = synonymous, The table shows any mutation present above 0.1 in at least one individual, and any mutation above 0.001 found in more than one individual.

**Fig 5:**
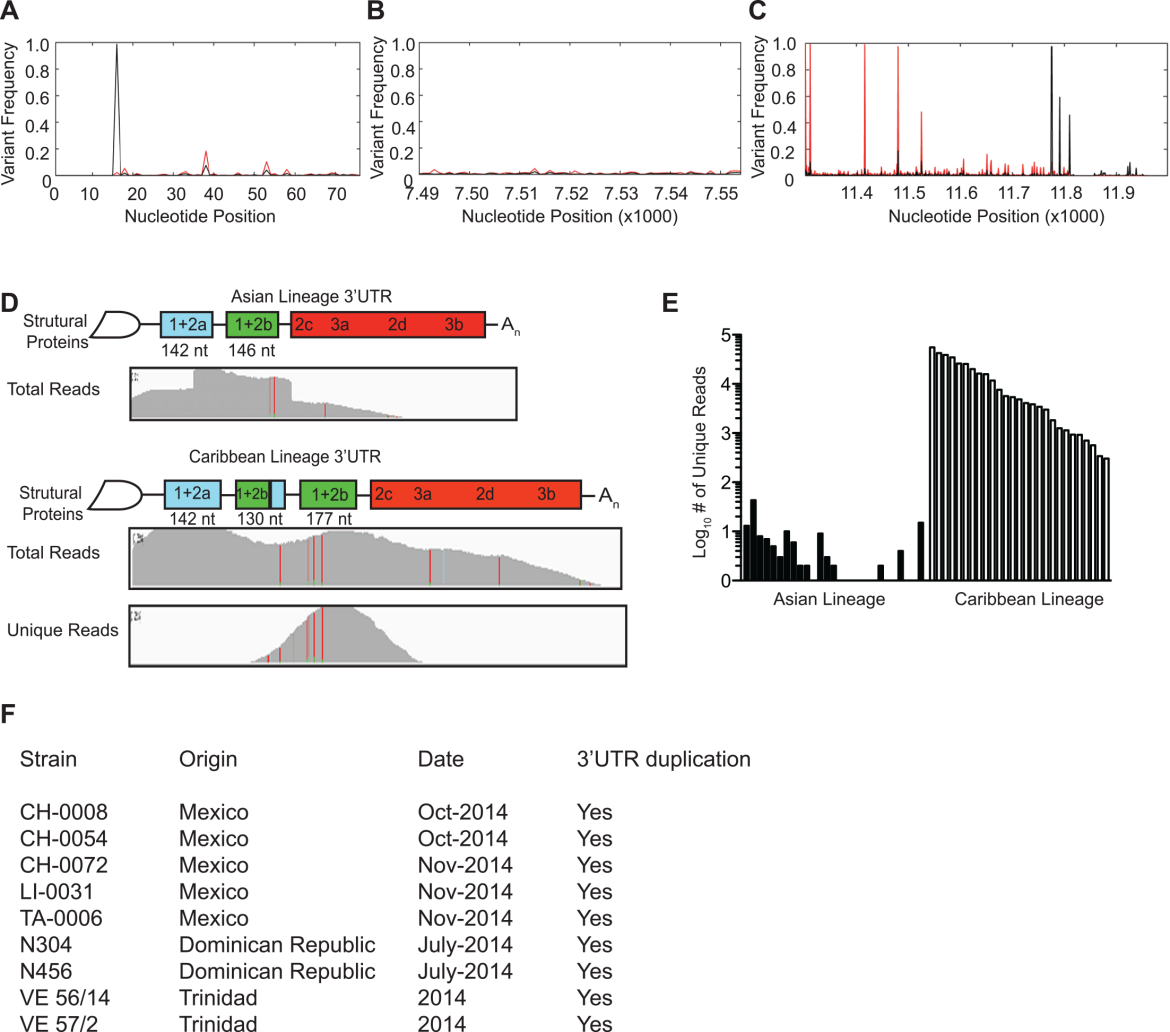
Analysis of minority variants in noncoding genome regions and identification of a novel duplication in the 3’UTR. A-C. Minority variants present in the 5’UTR, genome nucleotides 1–76 (A), subgenomic promoter, genome nucleotides 7490–7554 (B), and 3’UTR, genome sequence 11302–11944 (C). Graphs represent the mean variant frequency (black) and variant range (red) at each nucleotide. D. Schematic and alignment of the published Asian strain and novel Caribbean strain containing a 177 nucleotide duplication of the 3’ end of 1+2a and complete 1+2b in the 3’UTR. “Total reads” show the read coverage of all reads aligning to the two lineage references. E. Quantification of the total “unique reads”. Unique reads show the read coverage of all reads where the alignment to the Caribbean reference is superior to the alignment of the Asian Lineage. F. The presence of the 3'UTR duplication was confirmed in clinical samples obtained from Mexico [18], Dominican Republic and Trinidad.

In addition to characterizing the viral diversity present in the coding regions of the CHIKV genome, we also examined the diversity within the noncoding untranslated regions (UTR) (**Fig 5**). To begin, we analyzed the well-conserved 5’UTR (**Fig 5A**) and 3’ subgenomic promoter (**Fig 5B**) and found only slight variations in these regions (frequencies ranging from 0.01 to 10% in the 5’UTR and 0.01 to 1% in the subgenomic promoter), suggesting that RNA secondary structure in these regions is maintained and may be important for viral replication.

In contrast, we found a higher degree of genetic diversity within the 3’UTR. Interestingly, when we initially aligned the deep sequencing reads to the published St. Martin strain of CHIKV, we observed a near doubling of read coverage mapping to a region in the 3'UTR, spanning the 1+2a and 1+2b repeat regions (**Fig 5D**, Asian Lineage), suggesting that this region may have been duplicated. Examination of filtered reads that did not properly align to the original reference sequence identified reads that overlapped the expected junction site where the duplication would have occurred. Indeed, this 177 nucleotide duplication was confirmed by Sanger sequencing of RT-PCR amplicons and mapped to a duplication of the 3’ portion of the 1+2a region and complete 1+2b region (**Fig 5D**, Caribbean Lineage). Subsequently, when we generated a new reference sequence containing the expected duplication for alignment of deep-sequencing data, the reads that originally could not map to the genome now mapped perfectly to the duplication in all patients where sequence data were available (**Fig 5D and 5E**). This duplication was found in patients presenting both low and high viremia. Importantly, we confirmed this duplication by RT-PCR in CHIKV clinical samples from Mexico, the Dominican Republic and Trinidad (**Fig 5F)**, suggesting that this novel genetic element is present in samples throughout the Caribbean islands and Americas.

**Fig 6:**
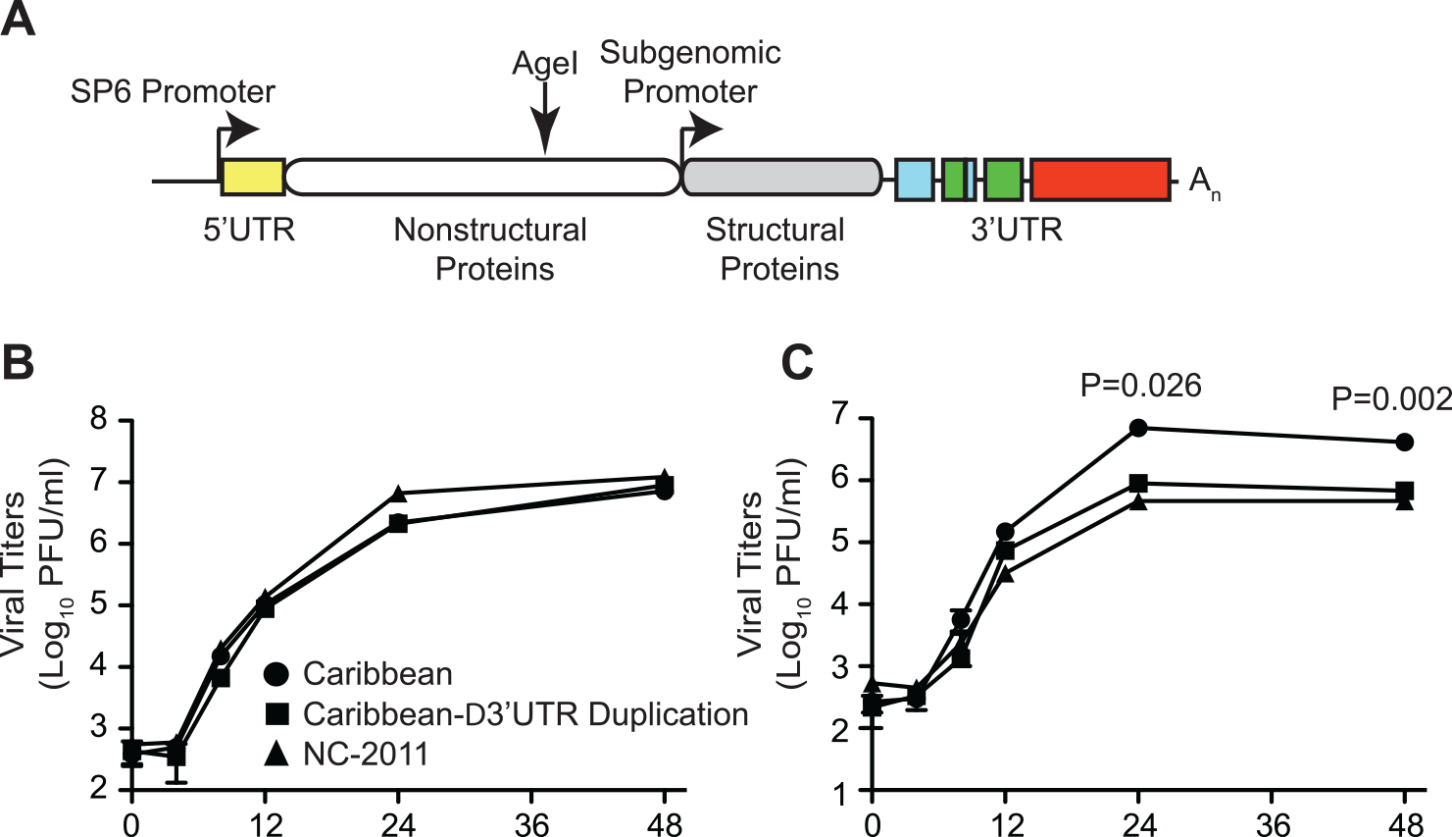
Design and growth of the Caribbean strain infectious clone. A. Schematic of the infectious clone, including a new synonymous AgeI restriction site and duplication in the 3’UTR. B and C. Viral growth curves of the Caribbean infectious clone (Caribbean–circle), a Caribbean clone lacking the 3’UTR duplication (Caribbean-∆3’UTR Duplication—square), or an Asian lineage strain of chikungunya virus (NC-2011—triangle) in either mammalian (Vero) or insect (C6/36) cells respectively. Mean values and SEM, n = 3, two-tailed unpaired t-test.

To understand the function and evolutionary potential of these novel minority variants and RNA structural elements, we constructed an infectious clone of this virus (**Fig 6A**) as well as obtained an infectious clone lacking the 3’UTR duplication (Caribbean-∆3’UTR Duplication). Similar to what has been published previously, we found both of these viruses to replicate similarly to another Asian lineage of CHIKV (NC-2011) in mammalian cells (**Fig 6B)** [19]. However, in mosquito cells we found the Caribbean strain containing the 3’UTR duplication to have a roughly 10-fold growth advantage over the Asian strain as well as a Caribbean strain lacking the 3’UTR duplication (**Fig 6C**). These data suggest that the 3’UTR duplication not only has no negative impact on viral replication, but that this novel element directly provides an advantage to Asian strains in insects. Prior historic duplications in the 3’UTR are believed to have allowed the Asian genotype to recover from genetic drift after its introduction from Africa in the late 19^th^ or early 20^th^ century [19]. This infectious clone, containing the correct 3'UTR of the American strain of CHIKV, will provide a powerful tool in which to study pathogenesis and evolution.

## Discussion

In this study we characterized the minority variants and viral populations within CHIKV-infected individuals from the recent Caribbean islands outbreak. It should be noted that the whole-genome analysis presented here was obtained from generally high viremia samples, which could possibly bias the observed diversity. Here we show that genetic diversity is spread throughout the coding region of the CHIKV genome, with higher levels of variation, including amino acid changes, in some non-structural proteins such as nsP3. Given the relatively low frequency of most of these variants (<10%), these mutations likely represent *de novo* generated neutral or lower fitness variants that would be purified during transmission bottlenecks. It is well known that the majority of mutations in RNA viruses bear a negative fitness cost [20] [21]. In our own study, human samples presented thousands of variants, over 80% of which had frequencies below 0.5%, yet above the background noise of sequencing. Most of the variants presenting stop mutations that would result in non viable virus are within this group. The variants presented here likely retained some level of replicative capacity, yet not sufficient fitness to outcompete the master sequence. It will be interesting to further characterize these variants in the context of the infectious clone, which we have developed to better understand how these variants may function in CHIKV pathogenesis and disease. Our data also indicate that a considerable loss of diversity present within human samples occurs after even a single passage in cell culture, especially in C6/36 mosquito cells. Even a single amplification in cell culture passage, although often necessary for diagnostics and surveillance, may thus introduce artifacts or purify previously existing minority variants that were in the process of emerging in human samples.

Phylogenetic analysis revealed that the strains collected in Guadeloupe and Martinique islands belong to the Asian genotype circulating in the Caribbean. Furthermore, the strains sequenced in this study show a closer phylogenetic relationship, which can be attributed to the short genetic distances depicted in their tree branches. We observed strains from Brazil (TR206 and AMA2798) and Mexico (InDRE_4CHIK and InDRE_51CHIK) to cluster with our cohort as well. Interestingly, the strain InDRE_4CHIK in Mexico was obtained from an imported case from Antigua and Barbuda, islands in close geographic proximity to Guadeloupe and Martinique [22] [23] and the strain from Brazil (TR206) was obtained from a patient who had recently traveled to Guadeloupe [15]. Finally, our phylogenetic analysis supports previous hypothesis about the introduction of CHIKV into the Americas by a single entry event of the Asian genotype [15].

Moreover, whereas previous analyses focused on amino acid changes that may impart a functional or enzymatic impact, we also analyzed variation in the noncoding, untranslated regions of the genome. In this analysis, we identified a novel 3’UTR duplication that had not previously been observed in nature, yet is present in several viruses circulating in the Americas including Mexico, the Dominican Republic and Trinidad. The fact that we have found this duplication in clinical samples from 2013 and again in 2015 suggests this duplication is fixed in the current circulating Caribbean strain. Importantly, we found this new UTR structure to provide no disadvantage for the virus and interestingly, it led to increases in viral titers in mosquito cells when compared to a similar Asian strain and a Caribbean strain clone lacking the duplication. As the 3’UTR has been shown to play essential roles in arbovirus replication, evolution, and host adaptation [19] [24], it will be interesting to dissect the role of this novel structure in its ability to specifically infect mosquitoes native to these affected areas as well as its ability to affect pathogenesis in humans.

These studies highlight the need to carefully re-analyze deep-sequencing assemblies, such as sudden increases in read coverage and the inability to map unfiltered reads that still contain virus-specific sequences, which may be indicative of duplications and insertions. The origins of this duplication still needs to be determined to understand if it first originated in Asia prior to arriving in the Caribbean, or was generated just before or after December 9, 2013, the start of the epidemic in St. Martin [25] and has since spread throughout the Americas due to a fitness advantage. These scenarios are both possible as chikungunya virus was first observed in Martinique on December 19, 2013 and Guadeloupe on December 28, 2013 and our earliest sample analyzed in this study was on December 26, 2013, shortly after its spread to Martinique. In either case, a population bottleneck was involved that may have facilitated the fixation of this beneficial insertion.

Nonetheless, this study provides an in depth look at the minority variants present during an ongoing chikungunya virus epidemic, identifying novel variants and structural elements, and the construction of an infectious clone of this virus to be used to future study the pathogenesis, adaptation and evolution of chikungunya virus in the Americas. The study of these evolutionary elements, which may play crucial roles in chikungunya virus evolution and adaptation, will be necessary to address potential future public health issues both in disease and viral dissemination.
